# Fatigue and Its Association With Quality of Life Among Carers of Patients Attending Psychiatric Emergency Services During the COVID-19 Pandemic

**DOI:** 10.3389/fpsyt.2021.681318

**Published:** 2021-06-22

**Authors:** Xiao Ji, Wen Li, Hui Zhu, Ling Zhang, Teris Cheung, Chee H. Ng, Yu-Tao Xiang

**Affiliations:** ^1^Beijing Key Laboratory of Mental Disorders, The National Clinical Research Center for Mental Disorders, The Advanced Innovation Center for Human Brain Protection, Beijing Anding Hospital, Capital Medical University, Beijing, China; ^2^Unit of Psychiatry, Department of Public Health and Medicinal Administration, Faculty of Health Sciences, Centre for Cognitive and Brain Sciences, University of Macau, Macao, China; ^3^Institute of Advanced Studies in Humanities and Social Sciences, University of Macau, Macao, China; ^4^School of Nursing, Hong Kong Polytechnic University, Hong Kong, China; ^5^Department of Psychiatry, The Melbourne Clinic, St Vincent's Hospital, University of Melbourne, Richmond, VIC, Australia

**Keywords:** psychiatric emergency service, fatigue, quality of life, COVID-19, carers

## Abstract

**Aims:** Carers of psychiatric patients often suffered from mental and physical burden during the coronavirus disease 2019 (COVID-19) pandemic due to the lack of mental health services. This study investigated the pattern of fatigue and its association with quality of life (QOL) among the carers of patients attending psychiatric emergency services during the COVID-19 pandemic.

**Methods:** In this cross-sectional study, carers of patients attending psychiatric emergency services during the COVID-19 pandemic were consecutively included. Fatigue, insomnia symptoms, depressive symptoms, and QOL were assessed with standardized instruments.

**Results:** A total of 496 participants were included. The prevalence of fatigue was 44.0% (95% CI = 39.6–48.4%). Multivariate logistic regression analysis revealed that fatigue was positively associated with higher education level (OR = 1.92, *P* < 0.01) and more severe depressive (OR = 1.18, *P* < 0.01) and insomnia symptoms (OR = 1.11, *P* < 0.01). ANCOVA analysis revealed that the QOL was significantly lower in carers with fatigue compared with those without (P = 0.03).

**Conclusions:** Fatigue was common among carers of patients attending psychiatric emergency services during the COVID-19 pandemic. Considering the adverse impact of fatigue on QOL and other health outcomes, routine screening and appropriate intervention for fatigue are warranted for this subpopulation.

## Introduction

As of the end of April 2021, the coronavirus disease 2019 (COVID-19), which was declared as a pandemic (1), has been reported in more than 200 countries/territories and caused more than 143 million confirmed cases and over 3 million deaths worldwide (2). Patients with psychiatric disorders have an increased risk of COVID-19 infection and mortality (3). In China, due to the lack of community mental health services, maintenance psychiatric treatments are mostly provided by outpatient departments of psychiatric hospitals, which are mostly located in urban areas (4, 5). During the COVID-19 pandemic, however, the lockdown restrictions and quarantine measures have prevented psychiatric patients from visiting psychiatric outpatient services, hindering their maintenance therapy (6). Moreover, visits to psychiatric outpatient departments may increase patients' exposure to nosocomial infections during the pandemic. For example, over 300 patients with psychiatric disorders were infected with the COVID-19 at the early stage of the COVID-19 outbreak in China (7). Hence, to reduce the risk of transmission, outpatient services in many psychiatric hospitals were restricted or suspended during the COVID-19 pandemic. Consequently, these factors have reduced the access to mental health services and maintenance treatment for psychiatric patients, which has led to more frequent illness relapses and presentations to psychiatric emergency services.

In China, the carers of psychiatric patients (i.e., spouse, parents, adult children, and close relatives and friends) are usually responsible for supervising their medication adherence at home. Additionally, the stigmatization, social discrimination, and economic burden of caring for their family member with psychiatric disorder, often lead to the carers' mental and physical distress (8–12). Furthermore, during the COVID-19 pandemic, the carer' burden has increased due to several patient factors such as impaired cognition, unhealthy lifestyle, poor self-care, and suboptimal health status, all of which could increase the risk of infection with COVID-19 in psychiatric patients compared with those without psychiatric disorders (13, 14). Therefore, their carers had to take more precautions to remind psychiatric patients about COVID-19-related information and supervise them to comply with the preventive measures. All the abovementioned challenges are likely to exacerbate carers' mental and physical distress.

Fatigue, a major stress-related symptom, is frequently experienced by carers of psychiatric patients (15). Fatigue is a multidimensional symptom, which involves subjective (e.g., feelings of tiredness, lack of energy, and decreased motivation), behavioral (e.g., decline in performance, such as inaccuracy and/or reaction time during a cognitive task), and physiological (e.g., alterations in brain activity) components (16). It is also associated with a range of negative outcomes, including poor work performance, negative emotions (17), and even increased risk of sudden death (18).

During the COVID-19 pandemic, fatigue was examined among healthcare professionals (19–22) and the general population (23). To date, no studies on fatigue in carers of psychiatric patients during the COVID-19 pandemic have been published. In addition, the association of fatigue and quality of life (QOL) in carers of psychiatric patients has not been studied. Quality of life, as a comprehensive health outcome (24, 25), reflects individuals' perception toward their multidimensional general well-being, including physical and mental health, social ability and relationships, personal expectations, and so forth (26). Hence, this study examined the patterns of fatigue and its association with QOL among the carers of patients attending psychiatric emergency services during the COVID-19 pandemic.

## Methods

### Participants

This cross-sectional study was conducted from April 30 to July 4, 2020 in the Emergency Department of Beijing Anding Hospital, which provided the only psychiatric emergency services for psychiatric patients in the Beijing municipality and neighboring provinces. The carers of patients who attended the psychiatric emergency service during the study period were consecutively invited to participate in the survey. The inclusion criteria were: (1) carers of patients with psychiatric disorders according to the International Statistical Classification of Diseases and Related Health Problems, 10th Revision (ICD-10) (27), (2) aged 18 years and above, and (3) being able to understand the content of the assessment and provide written informed consent before the survey. The study protocol was approved by the ethics committee of Beijing Anding Hospital.

Due to the risk of contagion, conventional face-to-face interviews could not be conducted. Following other studies (28–30), data collection was carried out using the WeChat-based survey program, “Questionnaire Star,” which has been commonly used in epidemiological surveys before and during the COVID-19 pandemic. WeChat is a social communication application for smartphones, with over 1 billion users in China including all the carers who were invited to participate in this study.

### Evaluation and Assessment Instruments

The assessments were completed in a consultation room. The basic sociodemographic and clinical characteristics, such as age, gender, education level, employment, average personal monthly income, major medical conditions, and preexisting psychiatric disorders, were recorded. The COVID-19 outbreak-related questions with “yes/no” options, including “concerns about the COVID-19 outbreak?” and “frequent use of mass media for COVID-19 related information,” were asked.

Severity of fatigue was evaluated with the “0–10” numeric rating scale (NRS) (31), with “0” indicating “no fatigue at all” and “10” indicating “unbearable fatigue.” A score of 4 and more indicated “having fatigue” (32, 33). Severity of insomnia symptoms (insomnia hereafter) was assessed using the Chinese version of Insomnia Severity Index (ISI) (34, 35), which was validated in Chinese populations (35). The ISI consisted of seven items with the total score ranging from 0 to 28. A higher total score indicated more severe insomnia. The severity of depressive symptoms (depression hereafter) was assessed using the Chinese version of the nine-item Patient Health Questionnaire (PHQ-9) (36, 37), which has been widely used in previous Chinese research (38). The total score of PHQ-9 ranged from 0 to 27 with a higher total score representing more severe depression. The overall QOL was evaluated with the total score of the first two items of the World Health Organization Quality of Life-Brief version (WHOQOL-BREF) (39–41), with a higher score indicating higher QOL.

### Data Analysis

All the data analyses were performed with the Statistic Package for Social Science (SPSS), Version 24.0. The P-P Plot was used to examine the normality of continuous variables. Univariate analyses were conducted to compare the sociodemographic and clinical data between carers with and without fatigue using two independent-sample *t*-tests, Mann–Whitney *U*-tests, and χ^2^ tests as appropriate. The independent associates of fatigue were tested using the binary logistic regression analysis with the “enter” method (i.e., all independent variables were included in the regression model at the same time regardless of whether the independent variables were significant or non-significant). In the logistic regression analysis, the sociodemographic and clinical variables with *P* < 0.1 in univariate analyses were entered as independent variables, while fatigue was the dependent variable. In addition, the independent association between fatigue and QOL was tested using the analysis of covariance (ANCOVA) with all variables with significant group differences in univariate analyses as covariates. The significance level was set at *P* < 0.05 (two-tailed).

## Results

Altogether, 505 carers of patients attending psychiatric emergency services during the COVID-19 pandemic were invited, of whom, 496 fulfilled the eligibility criteria and completed the assessment, giving a response rate of 98.2%. Their basic sociodemographic and clinical data are presented in Table 1. The mean age of the whole sample was 46.0 [standard deviation (SD) = 13.4] years, and 51.9% (*n* = 257) were males. The prevalence of fatigue was 44.0% [95% confidence interval (CI) = 39.6–48.4%), with a mean NRS score of 3.37 (SD = 2.70).

**Table 1:** The demographic and clinical characteristics of the participants and their association with fatigue.

**Variable**	**Total** ** (*****N*** **=** **495)**		**Not fatigue** ** (*****N*** **=** **277)**		**Fatigue** ** (*****N*** **=** **218)**		**Univariate analyses**			**Multivariate logistic regression**		
	***N***	**%**	***N***	**%**	***N***	**%**	**χ^**2**^**	**df**	***P***	***P***	**OR**	**95% CI**
Male gender	257	51.9	150	54.2	107	49.1	1.25	1	0.26			
Education level (college and above)	290	58.6	146	52.7	144	66.1	8.95	1	** <0.01**	** <0.01**	1.92	1.26–2.91
Employed	436	88.1	245	88.4	191	87.6	0.08	1	0.77			
Average monthly personal income (5,000 CNY^*^ and above)	244	49.3	131	47.3	113	51.8	1.00	1	0.31			
Major medical conditions	29	5.9	13	4.7	16	7.3	1.54	1	0.21			
Preexisting psychiatric disorders	22	4.4	5	1.8	17	7.8	10.31	1	** <0.01**	0.28	1.85	0.59–5.75
Concerns about COVID-19	338	68.3	184	66.4	154	70.6	1.00	1	0.31			
Frequent use of mass media for COVID-19 related information	282	57.0	161	58.1	121	55.5	0.34	1	0.55			
	**Mean**	**SD**	**Mean**	**SD**	**Mean**	**SD**	***t/Z***	**df**	***P***			
Age (years)	46.0	13.4	46.1	13.3	45.8	13.6	0.24	493	0.80			
PHQ-9 total score	2.8	5.2	1.0	2.3	5.2	6.7	−9.63	–^**#**^	** <0.01**	** <0.01**	1.18	1.08–1.29
ISI total score	3.8	5.4	1.9	3.0	6.3	6.7	−8.58	–^**#**^	** <0.01**	** <0.01**	1.11	1.03–1.19
Quality of life	6.7	1.6	7.2	1.5	6.1	1.6	7.74	493	** <0.01**			

# *Mann–Whitney U-tests*.

* *1 CNY ≈ 0.14 US$ during the investigation*.

*Bolded value: <0.05. CNY, Chinese Yuan; CI, confidence interval; COVID-19, coronavirus disease 2019; ISI, Insomnia Severity Index; OR, odds ratio; PHQ-9, nine-item Patient Health Questionnaire; SD, standard deviation*.

Univariate analyses revealed that carers with fatigue were more likely to have higher education level (*P* < 0.01), preexisting psychiatric disorders (*P* < 0.01), and more severe depression (*P* < 0.01) and insomnia (*P* < 0.01). The binary logistic regression analysis found that the fatigue was positively associated with higher education level [odds ratio (OR) = 1.92, 95% CI = 1.26–2.91, *P* < 0.01] and more severe depression (OR = 1.18, 95% CI = 1.08–1.29, *P* < 0.01) and insomnia (OR = 1.11, 95% CI = 1.03–1.19, *P* < 0.01). The ANCOVA analysis revealed that carers with fatigue had significantly lower QOL than those without [F _(1, 496)_ = 4.59, *P* = 0.03].

## Discussion

This was the first study that examined the pattern of fatigue among carers of patients attending psychiatric emergency services during the COVID-19 pandemic. We found that around half of the participants (44.0%) reported fatigue. The corresponding figure was 73.7% in Chinese frontline staff (including doctors, nurses, polices, volunteers, community workers, and journalists) during the COVID-19 pandemic (22). However, direct comparisons between studies should be made with caution due to different measurements of fatigue and sampling method.

A variety of reasons could contribute to the common occurrence of fatigue among carers of patients attending psychiatric emergency services during the COVID-19 pandemic. First, carer burden is increased during the COVID-19 pandemic as carers need to supervise patients to strictly comply with preventive and self-protection measures against the COVID-19. Second, lockdown restriction and quarantine measures as well as suspended/limited outpatient services in many hospitals could hinder or reduce access to psychiatric services, which could lead to illness relapse and increased carers' burnout and distress. Furthermore, quarantine measures could also limit carers' access to social support, such as friends and relatives who have previously helped with patient care. Third, the COVID-19 pandemic could have decreased employment opportunities and increased economic burden to the general population including the carers (42).

Fatigue was positively associated with depression in this study, which is consistent with studies in family caregivers in intensive care units (43), carers of patients with Parkinson's disease (44), and palliative care patients (45). The association between fatigue and depression is bidirectional. On one hand, fatigue could lead to negative emotions and increase the risk of depression (17, 46, 47). A longitudinal study over 13 years found that individuals with unexplained fatigue had greatly increased incidence of new episode of major depression compared with those without [risk ratio (RR) = 28.4, 95% CI = 11.7–68.0] (48). On the other hand, fatigue could also be caused by depression. It was reported that depressed patients often experience fatigue as a symptom, being one of the diagnostic criteria for major depression, which is reported in up to 87.2% of patients with major depression (49).

Previous studies have found that fatigue is one of the major consequences of insomnia (50, 51), which was confirmed in this study. With insufficient sleep, individuals may experience impaired executive function, which is associated with fatigue (52). Moreover, depression may mediate the correlation between fatigue and insomnia, as both fatigue and insomnia are key features of depression (53).

Among carers of patients with psychiatric disorders (54, 55) or physical illnesses (e.g., multiple sclerosis and cancer) (56–58), those with higher education level had more access to relevant information and had better coping patterns when caring for patients. Thus, they may have better psychological resilience and are less likely to experience health problems including fatigue. On the contrary, in this study, carers with higher education level reported more severe fatigue. We speculate that during the COVID-19 pandemic, carers with higher education level could have had more access to negative information on COVID-19, e.g., COVID-19 was repeatedly described as a “killer virus” in social media that perpetuated the sense of danger and uncertainty among the public, which may result in mental distress and fatigue.

As expected, carers with fatigue reported lower QOL than those without. Similar association was also reported among frontline staff during the COVID-19 pandemic (22). QOL is determined by the interaction between risk factors (e.g., mental and physical distress) and protective factors (e.g., good economic status and social support) based on the distress/protection QOL model (59). Fatigue is strongly associated with negative emotions [e.g., depression and anxiety (17, 46)], poor work performance, and poor physical health (18, 60, 61), all of which would lower QOL.

The strengths of this study included the relatively large sample size and use of standardized instruments. However, several methodological limitations need to be addressed. First, the causal relationship between fatigue and other variables could not be examined due to the cross-sectional design. Second, certain factors associated with fatigue and psychological burden in the carers [e.g., anxiety and somatization symptoms (22), severity and presence of physical diseases and relevant treatments, and illness duration and severity of psychiatric symptoms of the patients] were not assessed due to logistical reasons. Third, carers of patients were recruited from one psychiatric emergency service in Beijing, which limits the generalization of the findings to other areas of China.

In conclusion, fatigue is common among carers of patients attending psychiatric emergency services during the COVID-19 pandemic. Considering the adverse impact of fatigue on QOL and other health outcomes, routine screening and appropriate intervention for fatigue [e.g., occupational therapy (62), kinesiotherapy (63), interpersonal psychotherapy (64), and self-administered acupressure (65)] are warranted for this subpopulation.

## Data Availability Statement

The ethics committee of Beijing Anding Hospital that approved the study prohibits the authors from making the research dataset of clinical studies publicly available. Readers and all interested researchers may contact Dr. YT Xiang (Email address: xyutly@gmail.com) for details. Dr. Xiang could apply to the ethics committee of Beijing Anding Hospital for the release of the data.

## Ethics Statement

The studies involving human participants were reviewed and approved by the ethics committee of Beijing Anding Hospital. The patients/participants provided their written informed consent to participate in this study.

## Author Contributions

HZ and Y-TX designed the study. XJ, WL, and LZ collected, analyzed, and interpreted the data. XJ, WL, HZ, LZ, Y-TX, and TC drafted the manuscript. CN critically revised the manuscript. All authors contributed to the article and approved the submitted version.

## Conflict of Interest

The authors declare that the research was conducted in the absence of any commercial or financial relationships that could be construed as a potential conflict of interest. The reviewer JL declared a shared affiliation, though no other collaboration, with one of the authors, TC, to the handling editor.
